# Homocystinuria: A Rare Disorder Presenting as Cerebral Sinovenous Thrombosis

**Published:** 2015

**Authors:** Hossein ESLAMIYEH, Farah ASHRAFZADEH, Javad AKHONDIAN, Mehran BEIRAGHI TOOSI

**Affiliations:** Department of Pediatric Neurology, Mashhad University of Medical Sciences, Mashhad, Iran

**Keywords:** Cerebral sinovenous thrombosis, Homocystinuria, MRI, MRV

## Abstract

**Objective**

Homocystinuria is an inborn error of amino acid metabolism caused by cystathionine beta-synthase deficiency that affects methionine metabolism. The clinical features are heterogeneous ranging from mental retardation, ectopia lentis, and osteoporosis to vascular events such as deep vein thrombosis, sagital sinus thrombosis, and myocardial infarction. Cerebral sinovenous thrombosis (CVST) is an unusual disorder in children and requires prompt and accurate management. Some causal factors for the development of CVST differ between children and adults. The majority of cases with CSVT are found to have an underlying cause for thrombosis like dehydration, infections, prothrombotic and hematologic disorders, malignancy and trauma. Although homocystinuria is usually associated with ischemic strokes, CVST as initial clinical presentation of homocystinuria is rare in children. In this article, we presented a 10-year old boy with seizure, hemiparesis, and ataxia due to CSVT caused by homocystinuria.

## Introduction

Cerebral sinovenous thrombosis (CSVT), which is thrombosis within the cerebral venous system, occurs approximately in 1 in 100,000 children per year and accounts for 1 in 4 cases of childhood ischemic stroke (1). Thrombotic occlusion of cerebral sinuses causes rise in venous pressure with a consequence of developing pressure in cerebral tissue. This may occur focally in a single venous territory or more globally as a syndrome of diffuse increased intracranial pressure (2). Clinical presentations are often gradual and nonspecific. In contrast to acute ischemic stroke, diffuse neurological signs and symptoms are more common than focal deficits. They are more likely to develop over hours, days, or, even, weeks (3). Common clinical presentations include headache, lethargy, nausea, vomiting, and signs of increased intracranial pressure including papilledema and sixth nerve palsy. Seizures are more common in CSVT compared to acute ischemic stroke. In young infants, the presentation of disease is different from older children including seizures and diffuse encephalopathy. Diagnosis requires a high level of clinical suspicion and purposeful imaging of the cerebral venous system (4). The risk factors that produce thrombosis are definable in most children and frequently are multiple. They include head and neck infection, prothrombotic disorders, hematologic disorders, malignancy, trauma and systemic infection (5). Drugs, prothrombotic state, and common childhood illnesses including iron deficiency anemia and severe or even mild to moderate dehydration have been reported in CSVT (6, 7). Prothrombotic disorders are among the most common etiologies for CSVT and frequently detected in the childhood form. A number of specific prothrombotic disorders have been reported in pediatric CSVT including factor V Leiden, protein C or S deficiency, anticardiolipin antibodies, elevated lipoprotein, and homocystinuria. Homocystinuria is an unusual and treatable cause of CSVT (5). The catabolism of methionine produces cysteine with homocysteine as a pivotal intermediate. Most homocysteine is remethylated to methionine. This methionine-sparing reaction is catalyzed by the enzyme methionine synthase, which requires a metabolite of folic acid as a methyl donor and a metabolite of vitamin B12 as a cofactor. Further conversion of homocysteine to cystathionine requires a pyridoxal phosphate-dependent enzyme, cystathionine ß synthase, and the deficiency of which results in accumulation of homocysteine and reconversion of homocysteine to methionine (8). Homocystinuria type-1, due to the deficiency of cystathionine ß synthase, is the most common inborn error of methionine metabolism. It is characterized by ectopia lentis, progressive mental retardation, skeleton abnormalities like Marfan syndrome, and thromboembolic episodes. Diagnosis is usually made after 3 years of age when most patients present with subluxation of the lens (8, 9). The clinical features of homocystinuria are heterogeneous and range from mental retardation, ectopia lentis, and osteoporosis to vascular events such as deep vein thrombosis, sagital sinus thrombosis, and myocardial infarction (10). Although homocystinuria is usually associated with ischemic strokes, CVST as the initial clinical presentation of homocystinuria is rare in children (11) and to diagnose the underlying disorder, it needs to be recognized, because it is one of the preventable causes of CVST. In this article, we present a 10-year old boy with seizures, hemiparesis, and ataxia due to CSVT caused by homocystinuria.

## Case report

A 10 year-old boy referred with a chief complaint of seizure, left hemiparesis, and ataxia for 2 weeks. He was conscious on arrival to our center. His history is as follows: he was the second offspring of the parents and was born via normal vaginal delivery with a good APGAR. The parents were consanguineous and there were no problems in the neonatal period but he had a learning disorder in school. There was no history of previous signs of stroke. On physical examination, he was alert and did not have a fever. There were no signs or symptoms of meningeal irritation. He had Marfanlike features and because of history of ocular surgery for lens dislocation 2 years before, we could not detect papilledema. After admission, we requested brain magnetic resonance imaging (MRI) for him. The T2 weighted MRI revealed extensive areas of hyperintensity in the right parietal lobe and in the superior sagittal sinus. Hyperintensities in the left parietal lobe without right hemiparesis were suggestive of a previous stroke (Fig(s). 1, 2). We decided to have magnetic resonance venography (MRV) and thrombophilic tests done simultaneously, because we observed hyperintensity in the sagital sinus and the Marfanoid features of the patient. In the MRV, the superior sagittal sinus was obstructed (Fig(s). 3, 4) and in the thrombophilic investigations, the level of fasting plasma homocysteine was 245 mmol/l (the maximum normal range is 16.3 mmol/l) and methionine level was increased. Therefore, we established a diagnosis of homocystinuria. We stabilized the vital signs and initially treated the patient with phenytoin, heparin, and aspirin. After suspicion of homocystinuria, we administered pyridoxine, folic acid, vitamin B12, and betaine. After the initial treatment and rehabilitation, the signs and symptoms of hemiparesis and ataxia were progressively better.

## Discussion

Homocystinuria is a rare disorder in the metabolism of methionine. It leads to an abnormal accumulation of homocysteine and its metabolites in the blood and urine. The disease is inherited as autosomal recessive. In the classic disease cystathionine β-synthase, (CBS) activity is decreased or absent and homocysteine is not remethylated to methionine. Infants affected by this disorder are normal at birth. Clinical manifestations during infancy are nonspecific; include developmental delays, and a failure to thrive regardless of its manifestations, which were negligible (12). In homocystinuria, four organs demonstrate major involvement as follows: the central nervous system (CNS), skeletal, ocular, and vascular systems (1). Ocular manifestations usually occur after 3 years of age when subluxation of the ocular lens occurs. This causes severe myopia and iridodonesis. Subluxation of the lenses was detected at the age 8 years in our patient. Skeletal abnormalities resembling those of Marfan syndrome include thin and elongated limbs, scoliosis, high arch palate; pes cavus, and sometimes generalized osteoporosis are seen in the spinal X-ray. Our patient had, thin and elongated limbs, mild scoliosis, and high arch palate. CNS abnormalities have wide spectrum and IQ scores ranged from 10 to 130. Higher IQ scores are seen in vitamin B6 responsive patients (13). Progressive mental abnormalities are common with psychiatric and behavioral disorders observed frequently. Thromboembolic events involving large and small vessels are common and can occur at any age. These events occur especially in the brain vessels and result in ischemic stroke or cerebral sinovenous thrombosis. In our patient, the left hemiparesis was related to right parietal lobe stroke and seizures occurred later. All of these signs corresponded with homocystinuria (14). According to the focal neurological signs, an MRI was requested for him. A doubt for CSVT was introduced when hyperintensities observed in the superior sagittal sinus in T2 weighted, from this, an MRV was requested. The MRV showed obstruction of the superior sagittal sinus and diagnosis of CSVT was confirmed. In thrombophilic tests, all examinations were normal except for plasma amino acid chromatography, which showed significant elevated levels of homocysteine and methionine, and low levels of cysteine. Elevation of methionine and homocysteine in serum are diagnostic laboratory findings. Therefore, diagnosis of homocystinuria was confirmed and treatment with vitamin B6, folic acid, vitamin B12, and betaine was started. We recommend keeping in mind that every child with

**Fig 1:**
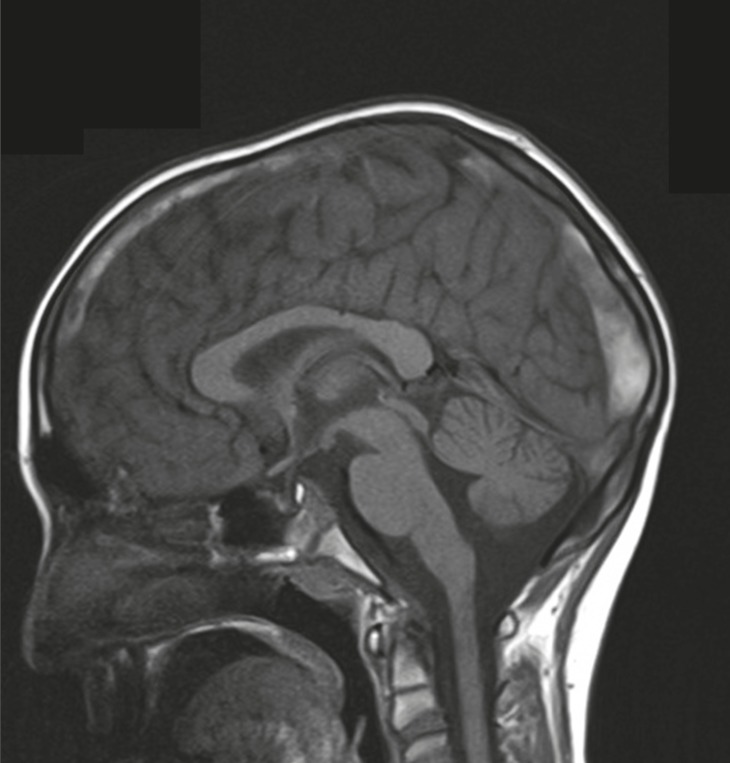
Sagital T1 view shows hyperintensity at the field

**Fig 2:**
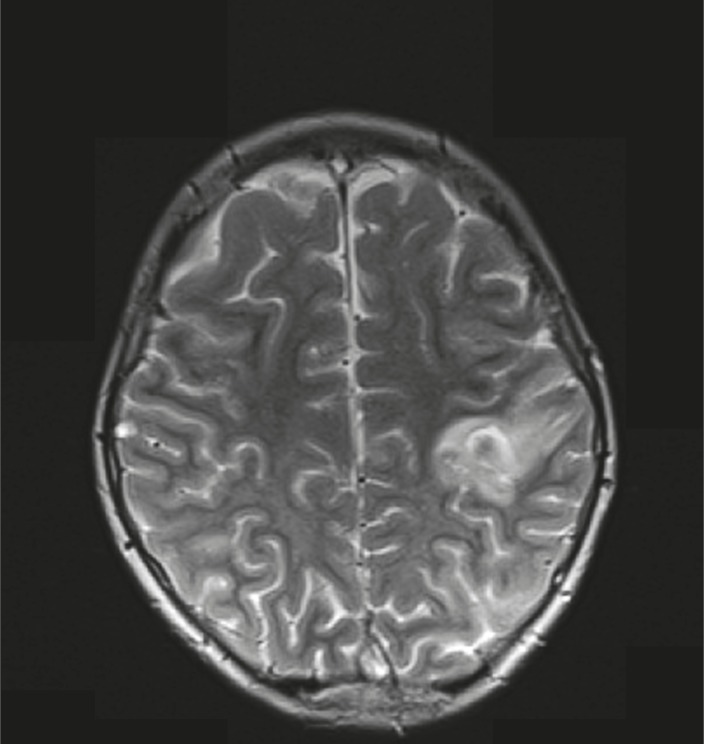
Axial T2 view shows bilateral hyperintensities in centrum-semi-ovals (with prominence in left parietal lobe) due to the sagital cerebral sinovenous thrombosis.

**Fig 3:**
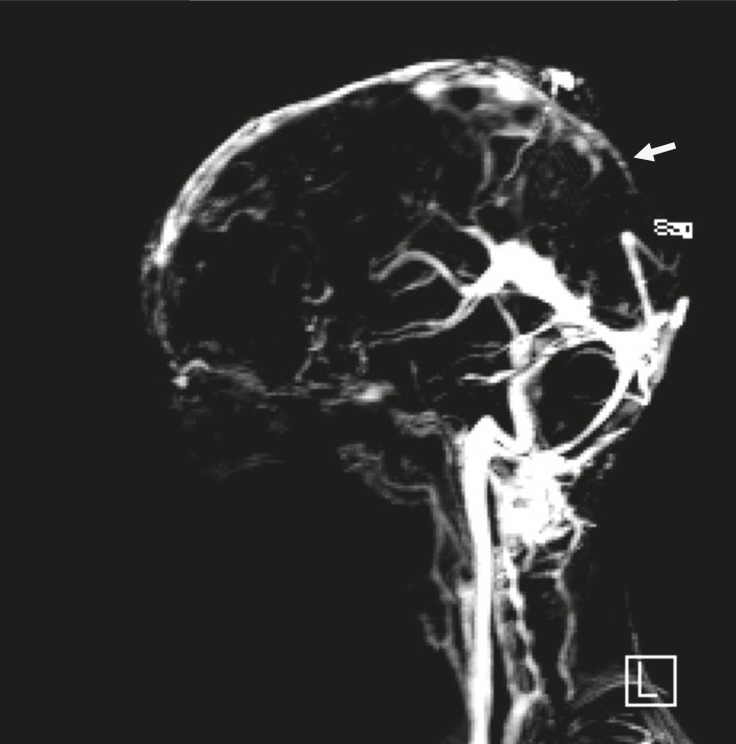
Sagital MRV view without gadolinium showing defect in sagital cerebral sinus (arrow).

**Fig 4:**
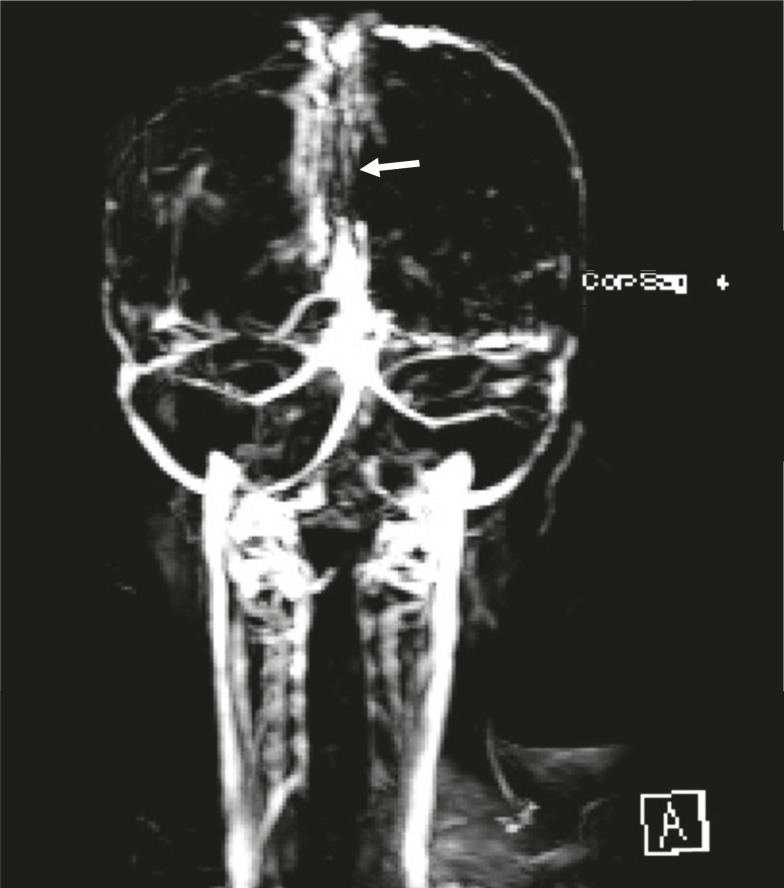
Coronal MRV view without gadolinium showing defect in sagital cerebral sinus (arrow).

CVST must be checked for homocystinuria, especially when other symptoms of the disease are present, because we could prevent later attacks with simple treatments.
